# Specimen collection is essential for modern science

**DOI:** 10.1371/journal.pbio.3002318

**Published:** 2023-11-22

**Authors:** Michael W. Nachman, Elizabeth J. Beckman, Rauri CK Bowie, Carla Cicero, Chris J. Conroy, Robert Dudley, Tyrone B. Hayes, Michelle S. Koo, Eileen A. Lacey, Christopher H. Martin, Jimmy A. McGuire, James L. Patton, Carol L. Spencer, Rebecca D. Tarvin, Marvalee H. Wake, Ian J. Wang, Anang Achmadi, Sergio Ticul Álvarez-Castañeda, Michael J. Andersen, Jairo Arroyave, Christopher C. Austin, F Keith Barker, Lisa N. Barrow, George F. Barrowclough, John Bates, Aaron M. Bauer, Kayce C. Bell, Rayna C. Bell, Allison W. Bronson, Rafe M. Brown, Frank T. Burbrink, Kevin J. Burns, Carlos Daniel Cadena, David C. Cannatella, Todd A. Castoe, Prosanta Chakrabarty, Jocelyn P. Colella, Joseph A. Cook, Joel L. Cracraft, Drew R. Davis, Alison R. Davis Rabosky, Guillermo D’Elía, John P. Dumbacher, Jonathan L. Dunnum, Scott V. Edwards, Jacob A. Esselstyn, Julián Faivovich, Jon Fjeldså, Oscar A. Flores-Villela, Kassandra Ford, Jérôme Fuchs, Matthew K. Fujita, Jeffrey M. Good, Eli Greenbaum, Harry W. Greene, Shannon Hackett, Amir Hamidy, James Hanken, Tri Haryoko, Melissa TR Hawkins, Lawrence R. Heaney, David M. Hillis, Bradford D. Hollingsworth, Angela D. Hornsby, Peter A. Hosner, Mohammad Irham, Sharon Jansa, Rosa Alicia Jiménez, Leo Joseph, Jeremy J. Kirchman, Travis J. LaDuc, Adam D. Leaché, Enrique P. Lessa, Hernán López-Fernández, Nicholas A. Mason, John E. McCormack, Caleb D. McMahan, Robert G. Moyle, Ricardo A. Ojeda, Link E. Olson, Chan Kin Onn, Lynne R. Parenti, Gabriela Parra-Olea, Bruce D. Patterson, Gregory B. Pauly, Silvia E. Pavan, A Townsend Peterson, Steven Poe, Daniel L. Rabosky, Christopher J. Raxworthy, Sushma Reddy, Alejandro Rico-Guevara, Awal Riyanto, Luiz A. Rocha, Santiago R. Ron, Sean M. Rovito, Kevin C. Rowe, Jodi Rowley, Sara Ruane, David Salazar-Valenzuela, Allison J. Shultz, Brian Sidlauskas, Derek S. Sikes, Nancy B. Simmons, Melanie L. J. Stiassny, Jeffrey W. Streicher, Bryan L. Stuart, Adam P. Summers, Jose Tavera, Pablo Teta, Cody W. Thompson, Robert M. Timm, Omar Torres-Carvajal, Gary Voelker, Robert S. Voss, Kevin Winker, Christopher Witt, Elizabeth A. Wommack, Robert M. Zink

**Affiliations:** 1 Museum of Vertebrate Zoology, UC Berkeley, Berkeley, California, United States of America; 2 Museum Zoologicum Bogoriense, National Research and Innovation Agency (BRIN), Cibinong, Indonesia; 3 Centro de Investigaciones Biológicas del Noroeste, La Paz, México; 4 Museum of Southwestern Biology, University of New Mexico, Albuquerque, New Mexico, United States of America; 5 Instituto de Biología, Universidad Nacional Autónoma de México, Mexico City, Mexico; 6 Museum of Natural Science and Department of Biological Sciences, Louisiana State University, Baton Rouge, Louisiana, United States of America; 7 Bell Museum of Natural History, University of Minnesota, Saint Paul, Minnesota, United States of America; 8 American Museum of Natural History, New York, New York, United States of America; 9 Field Museum of Natural History, Chicago, Illinois, United States of America; 10 Department of Biology, Villanova University, Villanova, Pennsylvania, United States of America; 11 Natural History Museum of Los Angeles County, Los Angeles, California, United States of America; 12 California Academy of Sciences, San Francisco, California, United States of America; 13 Biological Sciences, California State Polytechnic University, Humboldt, Arcata, California, United States of America; 14 Biodiversity Institute and Natural History Museum, University of Kansas, Lawrence, Kansas, United States of America; 15 Department of Biology, San Diego State University, San Diego, California, United States of America; 16 Departamento de Ciencias Biológicas, Universidad de los Andes, Bogotá, Colombia; 17 Biodiversity Center & Dept. of Integrative Biology, The University of Texas at Austin, Austin, Texas, United States of America; 18 Department of Biology, University of Texas at Arlington, Arlington, Texas, United States of America; 19 Natural History Museum and Dept. of Biology, Eastern New Mexico University, Portales, New Mexico, United States of America; 20 Museum of Zoology, University of Michigan, Ann Arbor, Michigan, United States of America; 21 Instituto de Cs. Ambientales y Evolutivas, Universidad Austral de Chile, Valdivia, Chile; 22 Museum of Comparative Zoology, Harvard University, Cambridge, Massachusetts, United States of America; 23 Museo Argentino de Ciencias Naturales “Bernardino Rivadavia", Buenos Aires, Argentina; 24 Natural History Museum of Denmark, University of Copenhagen, Copenhagen, Denmark; 25 Museo de Zoología, F.C. Universidad Nacional Autónoma de México, Mexico City, Mexico; 26 ISYEB, Muséum national d’Histoire naturelle, Paris, France; 27 Philip L. Wright Zoological Museum, University of Montana, Missoula, Montana, United States of America; 28 Biodiversity Collections and Dept. of Biological Sciences, University of Texas at El Paso, El Paso, Texas, United States of America; 29 Smithsonian Institution, National Museum of Natural History, Washington, DC, United States of America; 30 San Diego Natural History Museum, San Diego, California, United States of America; 31 Escuela de Biología, Universidad de San Carlos de Guatemala, Ciudad de Guatemala, Guatemala; 32 Australian National Wildlife Collection, CSIRO, Canberra, Australia; 33 New York State Museum, Albany, New York, United States of America; 34 Burke Museum, University of Washington, Seattle, Washington, United States of America; 35 Departamento de Ecología y Evolución, Universidad de la República, Montevideo, Uruguay; 36 Moore Laboratory of Zoology, Occidental College, Los Angeles, California, United States of America; 37 CONICET, Centro de Ciencia y Técnica Mendoza, Mendoza, Argentina; 38 University of Alaska Museum, Fairbanks, Alaska, United States of America; 39 National University of Singapore, Singapore; 40 Museo de Zoología, Pontificia Universidad Católica del Ecuador, Quito, Ecuador; 41 Unidad de Genómica Avanzada, Cinvestav, Mexico; 42 Museums Victoria Research Institute, Melbourne, Australia; 43 Australian Museum Research Institute, Australian Museum, Sydney, Australia; 44 Facultad de Ciencias de Medio Ambiente, Universidad Indoamérica, Quito, Ecuador; 45 Dept. of Fisheries, Wildlife & Conservation Sciences, Oregon State University, Corvallis, Oregon, United States of America; 46 Natural History Museum, London, United Kingdom; 47 North Carolina Museum of Natural Sciences, Raleigh, North Carolina, United States of America; 48 Friday Harbor Laboratories, University of Washington, Friday Harbor, Washington, United States of America; 49 Universidad del Valle, Cali, Colombia; 50 Dept. Ecology and Conservation Biology, Texas A&M University, College Station, Texas, United States of America; 51 University of Wyoming Museum of Vertebrates, University of Wyoming, Laramie, Wyoming, United States of America; 52 University of Nebraska State Museum, Lincoln, Nebraska, United States of America

## Abstract

Natural history museums are vital repositories of specimens, samples and data that inform about the natural world; this Formal Comment revisits a Perspective that advocated for the adoption of compassionate collection practices, querying whether it will ever be possible to completely do away with whole animal specimen collection.

In a recent Perspective, Byrne [1] emphasized that natural history museums “are essential hubs for research and education” but that their mission should be reimagined to focus on nonlethal collecting. We endorse many of the practices advocated by Byrne, including the storage of tissues, recordings, photos, and other data; embracing new technologies such as massively parallel DNA sequencing, μCT scanning, and stable isotope analysis; and large-scale digitization of collections and associated metadata. Indeed, many of these practices are widely used by museums today. We also welcome the call to provide stable financial support to maintain and expand the infrastructure of existing collections. However, we do not support the call to use new technologies “to replace the need for whole animal bodies.” Byrne’s position overstates the potential of new technologies to replace specimen-based research and fails to acknowledge the importance of whole-organism–based research in building the foundations of modern biology and in continuing to promote new discoveries.

Our intention is not to address all the claims or ethical assumptions made by Byrne. We fully realize that collecting specimens is not necessary or desirable in certain circumstances, and we value the scientific contributions of researchers who choose not to collect whole animals. The importance and ethics of scientific collecting have been reviewed in many recent papers (e.g., [2–4]). Rather, our goal is to underscore the tremendous value of ongoing, whole-organism specimen collection by highlighting some of the key scientific and societal gains that arise from this research (Box 1).

**Box 1. The value of whole-organism specimen collection**

Whole-organism specimens enable many kinds of research that would be difficult or impossible to conduct in a comprehensive way with nonlethal samples such as recordings or photos. A few examples of research enabled by whole-organism specimens and their associated tissues and data illustrate the value of museum collections [2–13].

- Discovery and description of new species
- The origins and spread of infectious diseases
- Studies of environmental degradation such as the accumulation of microplastics and mercury in fish or DDT in eggshells
- Most research on endoparasites and small invertebrates (which constitute the majority of all animals)
- Research on morphology and physiology of whole organisms
- Studies of gene expression and epigenetic modifications in wild animals, including gene regulatory changes associated with adaptation to different environments
- Research that links genomic variation to phenotypic differences
- Studies of the biotic consequences of global change in the Anthropocene
- A global scientific resource for future studies and future technologies

## Documenting biodiversity

Most of the Earth’s biodiversity remains to be characterized, with an estimated 86% of species yet to be described [14]. Voucher specimens in the form of whole organisms are an essential part of species descriptions, providing a physical reference against which other individuals can be compared. Photographs, recordings, and DNA sequences do not individually or collectively provide the same quality of information, nor do they maximize the potential for linking genotype with phenotype. For example, as genomic data have become part of the standard taxonomic toolkit, discovery of cryptic or nearly cryptic species diversity is now routine. However, verification of these species requires intensive anatomical analyses that are impossible without whole-organism voucher specimens. Moreover, most animal species are small arthropods such as insects and mites, the majority of which cannot be found using nonlethal means and cannot be identified without microscopic examination [5]. Similarly, research on the endoparasites of most species is not possible without collection of whole organisms. Finally, understanding evolutionary processes often involves the study of large series of voucher specimens that document geographic, temporal, age, or sexual variation in specific traits. These studies all rely on the collection of whole organisms.

## Conservation of species

The International Union for Conservation of Nature (IUCN) assesses species once they are described. Thus, there is typically no mechanism to initiate conservation efforts prior to species descriptions. In addition, many conservation threats to individual species have been identified because of research conducted using combinations of modern and historical specimens. For example, the effects of DDT on the thinning of bird eggshells prompted the ban on the use of DDT as a pesticide, leading to the subsequent recovery of threatened species. This work, which was based on linking eggshell weight and thickness to chemical concentrations [6], could not have been carried out from photographs or eggshell fragments. Similarly, the timing and spread of the chytrid fungus pandemic that has driven worldwide declines of amphibian populations continues to be documented using both historical and recently collected museum specimens [7].

## Conservation of geographic regions

Documenting regional patterns of biodiversity from museum specimens has led to the creation of new national parks or protected areas in many regions of the world. For example, the most important biodiversity hotspot of East Africa, in the Udzungwa and Rubeho highlands of Tanzania, was discovered and documented through comprehensive collecting efforts, resulting in large investments in better management and the establishment of a national park [8]. Biodiversity documented through collections is also helping conservations efforts in Guatemala, Indonesia, the Philippines, and other countries. In such instances, the establishment of protected areas preserves far more individual organisms than were collected by researchers at these locations. Biodiversity is highest in the tropics where it is understudied and underrepresented in scientific collections, both locally and globally. Biodiversity is often highest in countries with limited resources for technologies such as massively parallel DNA sequencing or μCT scanning. Specimen collection is essential to document biodiversity in these critical regions, many of which face habitat destruction.

## Linking genotype to phenotype

Museum collections are repositories of phenotypic diversity. A central challenge of modern biology is to understand how genetic variation generates phenotypic differences. Whole-organism collections that preserve phenotypic diversity among many sampled individuals provide the opportunity to study how that diversity is generated and maintained. For example, the NSF-funded oVert (Open Exploration of Vertebrate Diversity in 3D) project uses CT scanning of approximately 20,000 museum specimens to provide high-resolution 3D representations of internal anatomy across diverse vertebrate taxa. However, this database captures only a limited portion of the variation in one lineage, and such databases will be improved in the future only by adding more whole-organism specimens. By contrast, when only DNA samples are collected in the field (e.g., by nonlethal collecting), it becomes impossible to associate genotypes with most types of phenotypic data, severely limiting the utility of DNA sequences for many types of future study.

## Identifying, monitoring, and predicting zoonotic pathogen emergence

Because the majority of emerging diseases in humans comes from animals, whole specimens that include frozen tissues are essential to identifying new pathogens, understanding pathogen circulation, spillover potential, and host immunology [9]. For example, deer mice were identified as the primary reservoir for a new hantavirus in the Southwestern United States in 1993, and the origin and spread of this virus was traced using tissues archived in 2 museums [10]. Museum specimens also allow future pathogen discovery [11]. Indeed, the recent SARS-CoV-2 pandemic has revealed a major gap in biosecurity infrastructure; the lack of biological samples across geographic regions and taxonomic groups prevents scientists from quickly and reliably identifying novel pathogens and their hosts. Ongoing specimen collection would help create a biorepository to prepare for future pandemics by enabling early detection and providing a framework for understanding spillover events [11].

## Providing a resource for future technologies

Natural history museums are engaged in research today in ways that were unimaginable when many of our institutions were founded. Specimens collected in the distant past have enabled research that utilizes novel technologies including DNA sequencing, stable isotope analysis, chemical and pollutant analysis, and μCT scanning. Just as past museum scientists could not imagine all the uses of specimens in the future, we cannot imagine the technologies that might be available a hundred years from now. It is only by continuing to provide complete voucher specimens with rich associated metadata that we will be able to empower discoveries using yet-to-be developed technologies by future generations of scientists.

## Establishing a baseline for the future

Environmental change in the Anthropocene, including climate change, land-use change, biological invasions, environmental contaminants, and habitat loss and degradation, is affecting many aspects of life on Earth. Comparisons of historical and modern museum specimens allow us to document and study the effects of global change on individual species and ecological communities [12]. Specimen collections in rapidly changing habitats like urban environments provide a means for understanding both ecological and evolutionary responses to land-use change and environmental degradation [13]. Similarly, museum specimens can reveal the time course over which contaminants and pollutants have become widespread [13]. As we move into a time of even greater climate transition and land-use change, there has never been a more pressing need for contemporary collections that allow comparisons to the past and also serve as a baseline for the future [4].

The contributions of whole-organism collecting listed above are not exhaustive but highlight some of the key reasons why specimen collecting continues to add value to science and to issues of societal importance including conservation, zoonotic pathogens, environmental pollutants, and numerous others. Although a few of these lines of inquiry could be pursued in a limited way without new collections or without whole organisms, most could not. We support the development of new technologies that increase the information obtained from museum specimens, but these should augment and not replace other methods. Specimen collection is still essential for modern science.

## Supporting information

S1 File Spanish translation of comment. (DOCX)
