# Supplementary material for: Specimen collection is essential for modern science
Source: PLoS Biol. 2023 Nov 22;21(11):e3002318. doi: 10.1371/journal.pbio.3002318 (PMC10664955; doi:10.1371/journal.pbio.3002318)
Supplement: S1 File — (DOCX) [file pbio.3002318.s001.docx]

Comentario Formal

La recolección de especímenes es esencial para la ciencia moderna

Michael W. Nachman^1*^, Elizabeth J. Beckman^1^, Rauri C.K. Bowie^1^, Carla Cicero^1^, Chris J. Conroy^1^, Robert Dudley^1^, Tyrone B. Hayes^1^, Michelle S. Koo^1^, Eileen A. Lacey^1^, Christopher H. Martin^1^, Jimmy A. McGuire^1^, James L. Patton^1^, Carol L. Spencer^1^, Rebecca D. Tarvin^1^, Marvalee H. Wake^1^, Ian J. Wang^1^, Anang Achmadi^2^, Sergio Ticul Álvarez-Castañeda^3^, Michael J. Andersen^4^, Jairo Arroyave^5^, Christopher C. Austin^6^, F. Keith Barker^7^, Lisa N. Barrow^4^, George F. Barrowclough^8^, John Bates^9^, Aaron M. Bauer^10^, Kayce C. Bell^11^, Rayna C. Bell^12^, Allison W. Bronson^13^, Rafe M. Brown^14^, Frank T. Burbrink^8^, Kevin J. Burns^15^, Carlos Daniel Cadena^16^, David C. Cannatella^17^, Todd A. Castoe^18^, Prosanta Chakrabarty^6^, Jocelyn P. Colella^14^, Joseph A. Cook^4^, Joel L. Cracraft^8^, Drew R. Davis^19^, Alison R. Davis Rabosky^20^, Guillermo D'Elía^21^, John P. Dumbacher^12^, Jonathan L. Dunnum^4^, Scott V. Edwards^22^, Jacob A. Esselstyn^6^, Julián Faivovich^23^, Jon Fjeldså^24^, Oscar A. Flores-Villela^25^, Kassandra Ford^7^, Jérôme Fuchs^26^, Matthew K. Fujita^18^, Jeffrey M. Good^27^, Eli Greenbaum^28^, Harry W. Greene^17^, Shannon Hackett^9^, Amir Hamidy^2^, James Hanken^22^, Tri Haryoko^2^, Melissa T.R. Hawkins^29^, Lawrence R. Heaney^9^, David M. Hillis^17^, Bradford D. Hollingsworth^30^, Angela D. Hornsby^27^, Peter A. Hosner^24^, Mohammad Irham^2^, Sharon Jansa^7^, Rosa Alicia Jiménez^31^, Leo Joseph^32^, Jeremy J. Kirchman^33^, Travis J. LaDuc^17^, Adam D. Leaché^34^, Enrique P. Lessa^35^, Hernán López-Fernández^20^, Nicholas A. Mason^6^, John McCormack^36^, Caleb D. McMahan^9^, Robert G. Moyle^14^, Ricardo A. Ojeda^37^, Link E. Olson^38^, Chan Kin Onn^39^, Lynne R. Parenti^29^, Gabriela Parra-Olea^5^, Bruce D. Patterson^9^, Gregory B. Pauly^11^, Silvia E. Pavan^13^, A. Townsend Peterson^14^, Steven Poe^4^, Daniel L. Rabosky^20^, Christopher J. Raxworthy^8^, Sushma Reddy^7^, Alejandro Rico-Guevara^34^, Awal Riyanto^2^, Luiz A. Rocha^12^, Santiago R. Ron^40^, Sean M. Rovito^41^, Kevin C. Rowe^42^, Jodi Rowley^43^, Sara Ruane^9^, David Salazar-Valenzuela^44^, Allison J. Shultz^11^, Brian Sidlauskas^45^, Derek S. Sikes^38^, Nancy B. Simmons^8^, Melanie L. J. Stiassny^8^, Jeffrey W. Streicher^46^, Bryan L. Stuart^47^, Adam P. Summers^48^, Jose Julian Tavera Vargas^49^, Pablo Teta^23^, Cody W. Thompson^20^, Robert M. Timm^14^, Omar Torres-Carvajal^40^, Gary Voelker^50^, Robert S. Voss^8^, Kevin Winker^38^, Christopher Witt^4^, Elizabeth A. Wommack^51^, Robert M. Zink^52^

^1^ Museum of Vertebrate Zoology, UC Berkeley, Berkeley, California, USA

^2^ Museum Zoologicum Bogoriense, National Research and Innovation Agency (BRIN), Cibinong, Indonesia

^3^ Centro de Investigaciones Biológicas del Noroeste, México

^4^ Museum of Southwestern Biology, University of New Mexico, Albuquerque, New Mexico, USA

^5^ Instituto de Biología, Universidad Nacional Autónoma de México, Mexico City, Mexico

^6^ Museum of Natural Science, Louisiana State University, Baton Rouge, Louisiana, USA

^7^ Bell Museum of Natural History University of Minnesota, Saint Paul, Minnesota, USA

^8^ American Museum of Natural History, New York, New York, USA

^9^ Field Museum of Natural History, Chicago, Illinois, USA

^10^ Department of Biology, Villanova University, Villanova, Pennsylvania, USA

^11^ Natural History Museum of Los Angeles County, Los Angeles, California, USA

^12^ California Academy of Sciences, San Francisco, California, USA

^13^ Biological Sciences, California State Polytechnic University, Humboldt, Arcata, California, USA

^14^ Natural History Museum, University of Kansas, Lawrence, Kansas, USA

^15^ Department of Biology, San Diego State University, San Diego, California, USA

^16^ Departamento de Ciencias Biológicas, Universidad de los Andes, Bogotá, Colombia

^17^ Biodiversity Collections & Dept. of Integrative Biology, The University of Texas at Austin, Texas, USA

^18^ Department of Biology, University of Texas at Arlington, Texas, USA

^19^ Natural History Museum and Dept. of Biology, Eastern New Mexico University, Portales, NM, USA

^20^ Museum of Zoology, University of Michigan, Ann Arbor, Michigan, USA

^21^ Instituto de Cs. Ambientales y Evolutivas, Universidad Austral de Chile, Valdivia, Chile

^22^ Museum of Comparative Zoology, Harvard University, Cambridge, Massachusetts, USA

^23^ Museo Argentino de Ciencias Naturales “Bernardino Rivadavia", Buenos Aires, Argentina

^24^ Natural History Museum of Denmark, University of Copenhagen, Copenhagen, Denmark

^25^ Museo de Zoología, F.C. Universidad Nacional Autónoma de México, Mexico City, Mexico

^26^ Muséum National d'Histoire Naturelle, Paris, France

^27^ Philip L. Wright Zoological Museum, University of Montana, Missoula, Montana, USA

^28^ Biodiversity Collections and Dept. of Biological Sciences, University of Texas at El Paso, Texas, USA

^29^ Smithsonian Institution, National Museum of Natural History, Washington DC, USA

^30^ San Diego Natural History Museum, San Diego, California, USA

^31^ Escuela de Biología, Universidad de San Carlos de Guatemala, Ciudad de Guatemala, Guatemala

^32^ Australian National Wildlife Collection, CSIRO, Australia

^33^ New York State Museum, Albany, New York, USA

^34^ Burke Museum, University of Washington, Seattle, Washington, USA

^35^ Departamento de Ecología y Evolución, Universidad de la República, Montevideo, Uruguay

^36^ Moore Laboratory of Zoology, Occidental College, Los Angeles, California, USA

^37^ CONICET, Centro de Ciencia y Técnica Mendoza, Argentina

^38^ University of Alaska Museum, Fairbanks, Alaska, USA

^39^ National University of Singapore, Singapore

^40^ Museo de Zoología, Pontificia Universidad Catolica del Ecuador, Quito, Ecuador

^41^ Unidad de Genómica Avanzada, Cinvestav, Mexico

^42^ Museums Victoria Research Institute, Melbourne, Australia

^43^ Australian Museum Research Institute & Centre for Ecosystem Science, Sydney, Australia

^44^ Facultad de Ciencias de Medio Ambiente, Universidad Indoamérica, Quito, Ecuador

^45^ Dept. of Fisheries, Wildlife & Conservation Sciences, Oregon State University, Corvallis, Oregon USA

^46^ Natural History Museum, London, United Kingdom

^47^ North Carolina Museum of Natural Sciences, Raleigh, North Carolina, USA

^48^ Friday Harbor Laboratories, University of Washington, Friday Harbor, Washington, USA

^49^ Universidad del Valle, Cali, Colombia

^50^ Dept. Ecology and Conservation Biology, Texas A&M University, College Station, Texas, USA

^51^ Museum of Vertebrates, University of Wyoming, Laramie, Wyoming, USA

^52^ University of Nebraska State Museum, Lincoln, Nebraska, USA

* mnachman@berkeley.edu

En una Perspectiva reciente, Byrne [1] recalcó que los museos de historia natural son “ejes esenciales para la investigación y la educación”, pero que su misión debe ser reimaginada para enfocarse en la recolección no letal de material biológico. Respaldamos muchas de las prácticas por las que aboga Byrne, incluyendo almacenar tejidos, grabaciones, fotografías y otros datos; adoptar nuevas tecnologías como la secuenciación de ADN masivamente paralela, las microtomografías y el análisis de isótopos estables; y digitalizar a gran escala las colecciones y sus metadatos asociados. De hecho, muchas de esas prácticas ya son ampliamente empleadas por los museos hoy en día. También celebramos el llamado a asegurar apoyo financiero estable para mantener y expandir la infraestructura de las colecciones existentes. Sin embargo, no respaldamos el llamado a emplear nuevas tecnologías “para reemplazar la necesidad de cuerpos completos de animales”. La posición de Byrne exagera sobre el potencial de las nuevas tecnologías para reemplazar la investigación basada en especímenes y no reconoce la importancia de la investigación basada en organismos enteros para construir las bases de la biología moderna y continuar promoviendo nuevos descubrimientos.

Nuestra intención no es abordar todos los argumentos ni las suposiciones éticas que hace Byrne. Comprendemos plenamente que recolectar especímenes no es necesario ni deseable en algunas circunstancias y valoramos las contribuciones científicas de los investigadores que deciden no recolectar animales completos. La importancia y la ética de la recolección científica han sido abordadas en muchos artículos recientes [e.g., 2-4]. En cambio, nuestra meta es enfatizar el enorme valor de la recolección continua de especímenes de organismos completos, resaltando algunas de las ganancias clave que surgen de este tipo de investigaciones para la ciencia y la sociedad (Caja 1).

**Documentar la biodiversidad**. La mayor parte de la biodiversidad de la Tierra no está caracterizada: se estima que el 85% de las especies aún no se ha descrito [5]. Los especímenes testigo (i.e. *vouchers*) correspondientes a organismos completos son una parte esencial de las descripciones de las especies, que brindan una referencia física con la cual se puede comparar otros individuos. Las fotografías, grabaciones y secuencias de ADN no proporcionan, por sí mismas ni en conjunto, la misma calidad de información y no maximizan el potencial de vincular el genotipo con el fenotipo. Por ejemplo, en la medida en que los datos genómicos se han convertido en un componente estándar de la caja de herramientas de los taxónomos, el descubrimiento de especies crípticas o casi crípticas es ahora rutinario. Sin embargo, la verificación de esas especies requiere análisis anatómicos profundos que no son posibles sin los cuerpos completos de los especímenes testigo. Además, la mayoría de especies animales corresponde a pequeños artrópodos como insectos o ácaros que no se pueden encontrar empleando medios no letales y no se pueden identificar sin estudios microscópicos [6]. De modo similar, la investigación sobre los endopárasitos de la mayor parte de las especies es imposible si no se recolectan organismos completos. Finalmente, comprender procesos evolutivos a menudo involucra el estudio de series grandes de especímenes testigo que permiten documentar la variación geográfica, temporal, de edad o sexual en rasgos específicos. Todos esos trabajos dependen de la recolección de organismos completos.

**Conservación de Especies**. La Unión Internacional para la Conservación de la Naturaleza (UICN) evalúa a las especies una vez han sido descritas. Por lo tanto, típicamente no existen mecanismos para iniciar esfuerzos de conservación antes de que las especies se describan. Además, muchas amenazas a la conservación de especies particulares se han identificado gracias a investigaciones basadas en el estudio conjunto de especímenes modernos e históricos. Por ejemplo, los efectos del DDT en la reducción del grosor de las cáscaras de los huevos de aves condujo a la prohibición del uso del DDT como pesticida, lo que luego llevó a la recuperación de poblaciones de especies amenazadas. Este trabajo, que se basó en conectar el peso y grosor de las cáscaras de los huevos con concentraciones de químicos [7], no podría haberse hecho con fotografías o fragmentos de cáscaras. De forma similar, la dispersión en el tiempo y el espacio del hongo quitridio causante de una pandemia que ha conducido a declives de las poblaciones de anfibios por todo el mundo continúa siendo documentada empleando tanto especímenes de museo antiguos como especímenes recolectados recientemente [8].

**Conservación de regiones geográficas***.* La documentación de patrones regionales de biodiversidad a partir de estudios de especímenes de museo ha conducido a crear nuevos parques nacionales o áreas protegidas en muchas regiones del mundo. Por ejemplo, el punto caliente de biodiversidad más importante del oriente de África, en las tierras altas de Udzungwa y Rubeho, Tanzania, fue descubierto y documentado mediante esfuerzos exhaustivos de recolecta científica. Dichos esfuerzos redundaron en inversiones considerables orientadas a un mejor manejo y el establecimiento de un parque nacional [9]. Documentar la biodiversidad mediante colecciones también está aportando a esfuerzos de conservación en Guatemala, Indonesia, Filipinas y muchos otros países. En dichas instancias, el establecimiento de áreas protegidas preserva muchos más organismos individuales que los que son recolectados por los investigadores en esas localidades. La biodiversidad es mayor en el trópico, donde está poco estudiada y escasamente representada en colecciones científicas tanto local como globalmente. Además, la biodiversidad a menudo es mayor en países con recursos limitados para emplear tecnologías como la secuenciación masiva de ADN o las microtomografías. La recolección de especímenes es esencial para documentar la biodiversidad en esas regiones críticas, muchas de las cuales se enfrentan a la destrucción de sus hábitats.

**Conexión entre genotipo y fenotipo**. Las colecciones de museo son repositorios de diversidad fenotípica. Un desafío central para la biología moderna es comprender cómo la variación genética genera diferencias en el fenotipo. Las colecciones de organismos completos que preservan la diversidad fenotípica que existe entre muchos individuos en una muestra brindan la oportunidad de estudiar cómo se genera y se mantiene esa diversidad. Por ejemplo, el proyecto oVert (Exploración Abierta de la Diversidad de Vertebrados en 3D), financiado por la NSF, emplea escaneos de ~20,000 especímenes de museo para generar representaciones tridimensionales en alta resolución de la anatomía interna de un grupo diverso de taxones de vertebrados. Sin embargo, esta base de datos captura sólo una porción limitada de la variación en un linaje y dichos repositorios de información podrán ser mejorados en el futuro únicamente mediante la adición de más especímenes de organismos completos. En contraste, cuando sólo se recolectan muestras de ADN en el campo (e.g. por medios no letales), es imposible asociar los genotipos con la mayoría de tipos de datos fenotípicos. Esto limita de forma severa la utilitad de las secuencias de ADN para muchos tipos de estudios futuros.

**Identificación, monitoreo y predicción de la emergencia de patógenos zoonóticos**. Puesto que la mayoría de enfermedades emergentes que afectan a los humanos proviene de otros animales, los especímenes completos que incluyen tejidos congelados son esenciales para identificar nuevos patógenos y para comprender la circulación de patógenos, el potencial de cambio de hospederos y la inmunología de los hospederos [10]. Por ejemplo, en 1993 se determinó que los ratones *Peromyscus* son el principal reservorio de un nuevo hantavirus en el sudoeste de los EEUU, y se elucidó el origen y dispersión de este virus empleando tejidos archivados en dos museos [11]. Los especímenes de museo también permiten el descubrimiento futuro de patógenos [12]. De hecho, la pandemia reciente de SARS-CoV-2 reveló que existe una enorme brecha en la infraestructura de bioseguridad: la falta de muestras biológicas a través de regiones geográficas y grupos taxonómicos impide que los científicos identifiquen de forma rápida y confiable los nuevos patógenos y sus hospederos. La recolección continua de especímenes ayudaría a crear un biorepositorio para prepararnos para futuras pandemias, permitiendo la detección temprana y ofreciendo un marco para entender los eventos de saltos de patógenos entre hospederos [12].

**Brindar recursos para las tecnologías del futuro**. Hoy en día, los museos de historia natural están involucrados en investigaciones de maneras que eran inimaginables cuando se fundaron muchas de nuestras instituciones. Los especímenes recolectados en el pasado distante han permitido hacer investigaciones que usan tecnologías novedosas que incluyen la secuenciación de ADN, el análisis de isótopos estables, estudios químicos y de contaminantes y las microtomografías. Así como los científicos de museo del pasado no podrían haber imaginado todos estos usos de los especímenes en el futuro, nosotros no podemos imaginar las tecnologías que estarán disponibles dentro de un siglo. Solamente si continuamos obteniendo especímenes testigo completos con metadatos asociados robustos podremos empoderar nuevos descubrimientos científicos por parte de generaciones futuras con tecnologías por desarrollar.

**Establecer una línea de base para el futuro***.* El cambio ambiental en el Antropoceno, incluyendo el cambio climático y en el uso de la tierra, las invasiones biológicas, la contaminación ambiental y la pérdida y degradación de hábitats, está afectando muchos aspectos de la vida en la Tierra. Las comparaciones de especímenes históricos y modernos nos permiten documentar y estudiar los efectos del cambio global sobre especies individuales y comunidades ecológicas [13]. Las colecciones de especímenes en ambientes que cambian rápido, como los entornos urbanos, representan un medio para entender las respuestas ecológicas y evolutivas a los cambios en el uso de la tierra y la degradación ambiental [14]. De forma similar, los especímenes de museo pueden revelar el intervalo de tiempo en el que los contaminantes y agentes de polución se han extendido de forma amplia [14]. Conforme nos movemos hacia un tiempo con transiciones climáticas y cambios en el uso del suelo mayores, nunca ha existido una necesidad más apremiante para contar con colecciones contemporáneas que permitan hacer comparaciones con el pasado y sirvan como líneas de base para el futuro [4].

La enumeración de las contribuciones de las colecciones de organismos completos que hemos hecho no es exhaustiva, pero resalta algunas de las razones clave por las cuales la recolección de especímenes continúa agregando valor a la ciencia y a asuntos de importancia para la sociedad incluyendo la conservación, los patógenos zoonóticos, los contaminantes ambientales y varios otros. Aunque algunas de estas líneas de pesquisa pueden abordarse de forma limitada sin colecciones nuevas o sin organismos completos, esto no es posible para la mayoría. Respaldamos el desarrollo de nuevas tecnologías que incrementan la información que se obtiene de los especímenes de museo, pero éstas deben potenciar y no reemplazar a otros métodos. La recolección de especímenes es aún esencial para la ciencia moderna.

Referencias

1. Byrne AQ. Reimagining the future of natural history museums with compassionate collection. PLoS Biology. 2023;21: e3002101.

2. National Academies of Sciences, Engineering, and Medicine. Biological Collections: Ensuring Critical Research and Education for the 21st Century. Washington, DC: The National Academies Press. 2020; https://doi.org/10.17226/25592

3. Winker K, Reed JM, Escalante P, Askins RA, Cicero C, Hough GE, Bates J. The importance, effects, and ethics of bird collecting. Auk. 2010;127: 90–695.

4. Rohwer VG, Rohwer Y, Dillman CB. Declining growth of natural history collections fails future generations. PLoS Biol. 2022;20: e3001613.

5. Mora C, Tittensor DP, Adl S, Simpson AGB, Worm B. How many species are there on Earth and in the ocean? PLoS Biol. 2011;9(8): e1001127.

6. Dombrow HE, Colville JF, Bowie RCK. Review of the genus *Amblymelanoplia* (Coleoptera: Scarabaeidae: Melolonthinae: Hopliini) with the description of ninety-three new species from South Africa and observations on its biogeography and phylogeny. Zootaxa. 2022; 5163:1–278.

7. Hickey JJ, Anderson DW. Chlorinated hydrocarbons and eggshell changes in raptorial and fish-eating birds. Science 1968;162: 271-273.

8. Cheng TL, Rovito S, Wake DB, Vredenburg VT. Coincident mass extirpation of neotropical amphibians with the emergence of the infectious fungal pathogen *Batrachochytrium dendrobatidis*. Proc Natl Acad Sci USA 2011;108: 9502–9507.

9. Rovero F, Menegon M, Fjeldså J, Collett L, Doggart N, Leonard C, et al. Targetted vertebrate surveys enhance the faunal importance and improve explanatory models within the Eastern Arc Mountains of Kenya and Tanzania. Diversity Distrib. 2014;20: 1438-1449.

10. Dunnum JL, Yanagihara R, Johnson KM, Armien B, Batsaikhan N, Morgan L, Cook JA. Biospecimen repositories and integrated databases as critical infrastructure for pathogen discovery and pathobiology research. PloS Neglected Tropical Diseases. 2017;11: e0005133.

11. Yates TL, Mills JN, Parmenter CA, Ksiazek TG, Parmenter RR, Vande Castle JR, Peters CJ. The ecology and evolutionary history of an emergent disease: hantavirus pulmonary syndrome: evidence from two El Niño episodes in the American southwest suggests that El Niño–driven precipitation, the initial catalyst of a trophic cascade that results in a delayed density dependent rodent response, is sufficient to predict heightened risk for human contraction of hantavirus pulmonary syndrome. Bioscience. 2002;52: 989–998.

12. Colella JP, Bates J, Burneo SF, Camacho MA, Carrion Bonilla C, Constable I et al. Leveraging natural history biorepositories as a global, decentralized, pathogen surveillance network. PLoS Pathogens. 2021;17(6): e1009583

13. Moritz C, Patton JL, Conroy CJ, Parra JL, White GC, Beissinger SR. Impact of a century of climate change on small-mammal communities in Yosemite National Park, USA. Science. 2008;322: 261–264.

14. Schmitt CJ, Cook JA, Zamudio KR, Edwards SV. Museum specimens of terrestrial vertebrates are sensitive indicators of environmental change in the Anthropocene. Phil. Trans. R. Soc. B. 2018;374: 20170387.

Caja 1

**El valor de las colecciones de organismos completos**

Los especímenes de organismos completos permiten muchos tipos de investigación que sería difícil o imposible hacer de forma exhaustiva con muestras no letales como grabaciones o fotografías. A continuación enumeramos algunos ejemplos de investigaciones que son posibles gracias a los especímenes de organismos enteros y sus tejidos y datos asociados, ilustrando el valor de las colecciones de museo [2-4, 6-14].

- Descubrimiento y descripción de especies nuevas
- El origen y dispersion de enfermedades infecciosas
- Estudios de degradación ambiental como la acumulación de microplásticos y mercurio en peces, o el DDT en cáscaras de huevos
- La mayoría de investigaciones sobre endoparásitos y pequeños invertebrados (que constituyen la mayoría de todos los animales)
- Investigación en la morfología y fisiología de organismos completos
- Estudios de expresión génica y modificaciones epigenéticas en animales silvestres, incluyendo cambios en la regulación genética asociados con la adaptación a ambientes diferentes
- Investigaciones que vinclulan la variación genómica con diferencias fenotípicas
- Estudios de las consecuencias bióticas del cambio global en el Antropoceno
- Un recurso científico global para próximos estudios y tecnologías del futuro
